# Supplementary material for: Fat-associated lymphoid clusters control local IgM secretion during pleural infection and lung inflammation
Source: Nat Commun. 2016 Sep 1;7:12651. doi: 10.1038/ncomms12651 (PMC5025788; doi:10.1038/ncomms12651)
Supplement: Supplementary Information — Supplementary Figure 1 [file ncomms12651-s1.pdf]

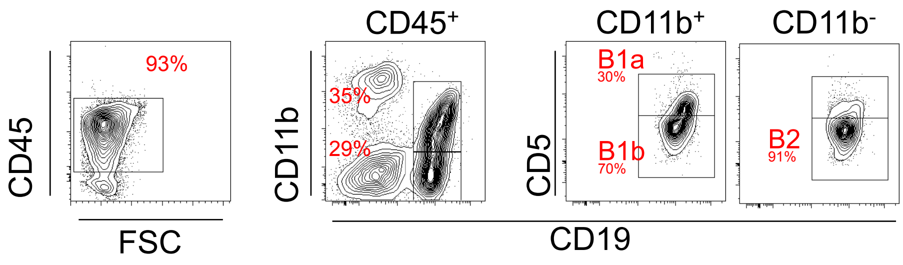

Supplementary Figure 1: B cell gating strategy

Representative flow cytometric gating strategy for B cell populations based on CD45, CD11b, CD5 and CD19.
